# Complete Genome Sequence of Mycobacterium orygis Strain 51145

**DOI:** 10.1128/MRA.01279-20

**Published:** 2021-01-07

**Authors:** Syed Beenish Rufai, Fiona McIntosh, Indira Poojary, Shubhada Chothe, Aswathy Sebastian, Istvan Albert, Craig Praul, Manigandan Venkatesan, Gibarni Mahata, Hindol Maity, Premanshu Dandapat, Joy Sarojini Michael, Robab Katani, Vivek Kapur, Marcel A. Behr

**Affiliations:** a Department of Microbiology and Immunology, McGill University, Montreal, Quebec, Canada; b McGill International TB Centre, Montreal, Quebec, Canada; c Infectious Diseases and Immunity in Global Health Program, Research Institute of the McGill University Health Centre, Montréal, Canada; d Huck Institutes of the Life Sciences, Pennsylvania State University, University Park, Pennsylvania, USA; e Department of Biochemistry and Molecular Biology, Pennsylvania State University, University Park, Pennsylvania, USA; f Department of Clinical Microbiology, Christian Medical College Vellore, Vellore, India; g Cisgen Biotech Discoveries, Chennai, India; h Indian Veterinary Research Institute, Eastern Region Station, Kolkata, West Bengal, India; i Department of Animal Science and the Huck Institutes of the Life Sciences, Pennsylvania State University, University Park, Pennsylvania, USA; University of Maryland School of Medicine

## Abstract

We report the complete 4,352,172-bp genome sequence of Mycobacterium orygis strain 51145 assembled into a single circular chromosome. Comparative genomic analyses with other lineages of the Mycobacterium tuberculosis complex can provide insights into the biology, evolution, and epidemiology of this important group of pathogenic mycobacteria.

## ANNOUNCEMENT

Within the Mycobacterium tuberculosis complex, M. orygis has received increasing recognition. Veterinary cases have been detected in a wide range of hosts, including oryxes, cattle, antelopes, and rhesus monkeys, and human cases have been reported mainly in patients of South Asian origin (1–6). Its detection among cattle in South Asia supports the possibility of a livestock reservoir, but the prevalence of M. orygis in these hosts is poorly understood (5, 6). Defining the genetic landscape of M. orygis is essential for understanding the biology and epidemiology of this human and veterinary pathogen.

M. orygis isolate 51145 was recovered from a human diagnosed with tuberculous meningitis in 1997 and initially classified by the Laboratoire de Santé Publique du Québec as M. caprae based on phenotypic characteristics (MPT70 positive, pyrazinamide sensitive). In 2005, the sample was referred to the McGill University Health Centre, Montreal, Canada, where it was definitively identified, based on specific genomic deletions, as “oryx bacillus,” the name given for M. orygis at that time (7, 8). To characterize the genome, M. orygis 51145 cultures were revived from frozen glycerol stocks and grown to mid-log phase in Middlebrook 7H9 (Difco, Becton Dickinson) with oleic acid, albumin, dextrose, and catalase (OADC) enrichment and polymyxin B, amphotericin B, nalidixic acid, trimethoprim, and azlocillin (PANTA). DNA extraction was performed using a MagAttract high-molecular-weight (HMW) DNA kit (Qiagen, Inc., Valencia, CA), and the quality of DNA was checked using a Quant-iT Pico Green double-stranded DNA (dsDNA) assay kit (Thermo Fisher Scientific) (9).

Whole-genome sequencing was performed using PacBio single-molecule real-time (SMRT) sequencing technology at the Penn State Genomics Core Facility, University Park, Pennsylvania. Briefly, DNA was sheared to an average size of 10 kb using a Covaris g-TUBE for preparation of SMRTbell libraries without further DNA size selection. The template was prepared using the SMRTbell Express template prep kit 2.0 according to the manufacturer’s protocol (PacBio, Menlo Park, CA). Sequencing was performed on a PacBio Sequel instrument using v3.0 chemistry and a 20-hour movie time. Circular consensus sequences (CCS) were generated from subreads with at least three full passes to achieve Q20 read accuracy. A total of 25,135 CCS were generated with average and maximum read lengths of 2,263 bp and 11,835 bp, respectively. CCS reads were corrected, trimmed, filtered, and *de novo* assembled (genome Size = 5 m and min Overlap Length = 100) using Canu v2.0, which generated 15 contigs (8). Assembled contigs were merged and circularized with Circlator v1.5.5 using default parameters, generating two scaffolds (10). The remaining gap was closed by reference-guided alignment of subreads to the M. bovis genome (AF2122) and identification of subreads that bridged the two scaffolds, resulting in a single contig of 4,352,172 bp. Annotation was performed using the NCBI Prokaryotic Genome Annotation Pipeline (11). The M. orygis 51145 chromosome size is 4,352,172 bp with a GC content of 65.6%. It encodes 4,039 genes and 45 tRNA and 3 rRNA sequences.

### Data availability.

The Mycobacterium orygis 51145 genome was deposited in GenBank under the accession number CP063804 and BioProject accession number PRJNA672657. The version outlined here is the first version of the accession number. CCS raw reads and subreads have been made available in the Sequence Read Archive database under SRA accession numbers SRR12966358 and SRR12916665.
